# Metformin for Treating Gestational Diabetes: What Have We Learned During the Last Two Decades? A Systematic Review

**DOI:** 10.3390/life15010130

**Published:** 2025-01-20

**Authors:** Angeliki Gerede, Ekaterini Domali, Christos Chatzakis, Chrysoula Margioula-Siarkou, Stamatios Petousis, Sofoklis Stavros, Konstantinos Nikolettos, Evanthia Gouveri, Sotirios Sotiriou, Panagiotis Tsikouras, Konstantinos Dinas, Nikolaos Nikolettos, Nikolaos Papanas, Dimitrios G. Goulis, Alexandros Sotiriadis

**Affiliations:** 1 Unit of Maternal-Fetal-Medicine, Department of Obstetrics and Gynecology, Medical School, Democritus University of Thrake, 67100 Komotini, Greece; knikolet@med.duth.gr (K.N.); ptsikour@med.duth.gr (P.T.); nnikolet@med.duth.gr (N.N.); 2 First Department of Obstetrics and Gynecology, Medical School, National and Kapodistrian University of Athens, 11527 Athens, Greece; kdomali@yahoo.fr; 3 Second Department of Obstetrics and Gynecology, Medical School, Aristotle University of Thessaloniki, 54124 Thessaloniki, Greece; cchatzakis@gmail.com (C.C.); margioulasiarkouc@gmail.com (C.M.-S.); petousisstamatios@gmail.com (S.P.); konstantinosdinas@hotmail.com (K.D.); asotir@gmail.com (A.S.); 4 Third Department of Obstetrics and Gynecology, Medical School, National and Kapodistrian University of Athens, 11527 Athens, Greece; sfstavrou@med.uoa.gr; 5 Diabetes Centre, Second Department of Internal Medicine, University Hospital of Alexandroupolis, Democritus University of Thrace, 68100 Alexandroupolis, Greece; vangouv@yahoo.gr (E.G.); papanasnikos@yahoo.gr (N.P.); 6 Department of Embryology, Faculty of Medicine, School of Health Sciences, University of Thessaly, 41334 Larissa, Greece; sotirious@med.uth.gr; 7 Unit of Reproductive Endocrinology, First Department of Obstetrics and Gynecology, Medical School, Aristotle University of Thessaloniki, 54124 Thessaloniki, Greece; dgg@auth.gr

**Keywords:** metformin, gestational diabetes mellitus, gestational weight gain, glycemic control

## Abstract

There has been accumulating evidence over the past two decades that metformin can be an effective treatment for gestational diabetes mellitus (GDM) in women whose diet and exercise fail to attain optimal glycemic control. The objective of this review was to comprehensively analyze all studies investigating the effectiveness of metformin compared to insulin and other drugs utilized for the treatment of GDM. After a comprehensive literature review based on PRISMA 2020, 35 studies were included after a selection process utilizing predetermined inclusion and exclusion criteria. A variety of short-term maternal and neonatal outcomes were assessed. Metformin is a highly efficient medication for attaining optimal control of blood sugar levels in women with GDM, resulting in a significant reduction in the amount of weight gained during pregnancy. Regarding additional maternal outcomes, such as pregnancy-induced hypertension and cesarean deliveries, some studies demonstrate a link between metformin and a reduced occurrence of both conditions. In contrast, others do not find an association. Regarding short-term neonatal outcomes, metformin does not exhibit any changes in gestational age at delivery. In contrast, metformin demonstrated substantial decreases in the likelihood of greater gestational birth weight and neonatal hospitalization when compared to other drugs. When compared primarily to insulin, metformin decreases the probability of several short-term outcomes related to pregnancy and newborns. Additional data are necessary for extended follow-up studies, including patients with GDM treated with metformin.

## 1. Introduction

Insulin resistance increases gradually during pregnancy due to complex hormonal changes [1] through mechanisms not completely clarified [2,3]. However, insulin resistance has been associated with several pregnancy-related hormones, including estrogens, free cortisol, human placental lactogen, and plasma progesterone, detected in high concentrations in maternal blood [4]. The increase in insulin resistance causes an increase in glucose synthesis and, simultaneously, a reduction in glucose absorption and metabolism to provide the embryo with the energy required for its development [5]. However, these physiologic alterations in the maternal metabolism may lead to gestational diabetes mellitus (GDM), which is characterized by glucose intolerance of variable severity and short- and long-term negative impacts on maternal and fetal health [6]. Nearly 14% of all pregnancies are complicated by GDM [7]. GDM has been associated with various adverse maternal and fetal outcomes, including pre-eclampsia, eclampsia, premature delivery, cesarean delivery (CD), macrosomia, neonatal hypoglycemia, neonatal respiratory distress syndrome (RDS), and an elevated risk of developing type 2 diabetes mellitus (T2DM), obesity, and cardiovascular disease (CVD) in the mother after pregnancy [8,9].

To highlight its potentially harmful effects on human health, the World Health Organization released a clinical guideline in 2018, in which GDM was officially designated a “global health research priority” [10]. GDM can present in three phenotypes: fasting hyperglycemia, postprandial hyperglycemia, and mixed hyperglycemia [11]. Obesity, nutritional deficiencies, a family history of insulin resistance or diabetes mellitus, and advanced maternal age are factors that contribute to an increased GDM risk [12]. These mothers may develop T2DM 5 to 10 years after delivery, experience obstructed labor, develop hypertension, and give birth to large-for-gestational-age (LGA) babies [7,13,14]. In certain instances, the higher increase in insulin resistance occurs in individuals who exhibit insulin resistance pre-pregnancy, such as patients with obesity, polycystic ovarian syndrome (PCOS), or T2DM [15,16,17].

Elevated maternal and fetal plasma glucose concentrations can be detrimental to the fetus. Complications may include premature fetal death, congenital anomalies, macrosomia, and potentially long-term complications for the offspring [18,19]; indeed, the novel notion of “metabolic memory” posits that hyperglycemia during pregnancy may influence the fetal hypothalamus in a way that programs adult progeny to develop obesity and metabolic syndrome [20].

Metformin is a long-standing and extensively utilized oral biguanide medication for controlling high blood glucose concentrations by decreasing glucose production by the liver, enhancing the liver’s insulin sensitivity, improving the transportation of glucose in muscles, and decreasing fat accumulation in the liver [8,21,22]. It is commonly prescribed independently or with other medications, such as insulin [23,24,25]. Furthermore, its distinctive features may enable its application in other pregnancy-related conditions, including breastfeeding, fertilization and PCOS. It is even suggested for pregnant women with obesity without T2DM [21]. However, the molecule crosses the placenta, achieving high concentrations in the fetal circulation [26,27,28,29,30].

The potential value of this medication is further highlighted since metformin has been demonstrated to have anticancer effects through a variety of mechanisms, such as the activation of ATM and ATM targets, the inhibition of lipogenesis in malignant lesions, and the modulation of mTOR activity [31,32]. These mechanisms result in a decrease in metabolic activity and the regulation of cellular senescence, which could be essential for the prevention of tumorigenesis [33,34,35,36]. Metformin has demonstrated potential as a treatment for specific cancer types, with certain studies suggesting favorable outcomes in patients with colon, rectum, pancreas, breast, prostate or liver cancers [32,35]. Meta-analyses and observational studies consistently show that metformin has the potential to serve as a preventive agent by reducing the incidence of cancer and mortality. These results underscore the extensive array of anticancer effects that metformin possesses, which extend beyond its primary application in the treatment of T2DM [31,36,37,38]. Ongoing clinical trials offer hope for the clarification of the function of metformin in cancer therapy and the refinement of treatment strategies [31].

Even though several observational studies and randomized controlled trials (RCTs) support the safety of metformin for GDM treatment, the US Food and Drug Administration characterizes it as a “category B” medication, meaning that relative animal reproduction studies have failed to demonstrate a risk to the fetus [8,26,39].

According to worldwide guidelines, the American Diabetes Association (ADA) and the American College of Obstetricians and Gynecologists (ACOG) suggest metformin as a secondary choice for the treatment of GDM [39,40,41,42]. The International Federation of Gynecology and Obstetrics (FIGO) recommends insulin as the first-line option for GDM, with metformin being an alternative, second-line option in specific cases [43].

In 2020, the National Institute for Health and Care Excellence (NICE) was the first to recommend metformin as a first-line treatment for GDM in their clinical guidelines. Insulin is advised as an alternative when metformin cannot be used due to contraindications [44]. On the other hand, in 2023, the Italian Association of Medical Diabetologists, the Italian Society of Diabetology, and the Italian Study Group of Diabetes in pregnancy recommended that metformin might be used for GDM as a second-line option or, in addition to insulin, reducing its dosage, particularly in obese women [45]. A review of the literature reveals a lack of consensus regarding the use of metformin to treat GDM.

Despite metformin’s seemingly established maternal safety, persistent worries remain about its possible adverse effects due to the drug’s placental crossing. This review aims to consolidate all the research findings from randomized controlled trials (RCTs) and systematic reviews and/or meta-analyses (SRs/MAs) conducted in the past 20 years to help physicians determine the most effective form of metformin usage for treating GDM.

## 2. Materials and Methods

### 2.1. Study Design

Using a systematic and exhaustive methodology, this study compared the effectiveness and safety of metformin against insulin and other drugs for treating gestational diabetes mellitus. A thorough exploration of the literature was conducted to identify relevant studies, followed by a rigorous selection process based on predefined inclusion and exclusion criteria.

### 2.2. Literature Search

A comprehensive electronic literature search was performed on multiple open-access databases, such as PubMed, Embase, Web of Knowledge, Scopus, Clinical Trial Registries, and the Cochrane Library. The search utilized a carefully selected collection of terms, such as “GESTATIONAL DIABETES MELLITUS”, “METFORMIN”, “INSULIN”, “GLIBENCLAMIDE”, “RANDOMIZED CONTROLLED TRIALS”, “DIABETES”, “GESTATIONAL DIABETES PREGNANCIES”, and “GESTATIONAL DIABETES.” The search technique was customized for each database, ensuring a uniform overall structure. The search was restricted to research published in English from 1 January 2004 to 29 July 2024, excluding editorials, conference papers, letters, and comments.

### 2.3. Studies Selection and Eligibility

Inclusion Criteria:

- Participants: pregnant women diagnosed with gestational diabetes mellitus (GDM) according to the International Association of Diabetes and Pregnancy Study Groups (IADPSGs) criteria or equivalent national guidelines, and women with varying degrees of GDM severity (e.g., mild, moderate, severe).
- Study Design: randomized controlled trials (RCTs) with adequate randomization and blinding procedures, cohort studies with appropriate matching or statistical adjustments for potential confounders, and studies with a minimum sample size to ensure adequate statistical power.
- Intervention:
  ○ Comparison of metformin therapy with insulin therapy (various regimens: basal-bolus, multiple daily injections, etc.); other oral hypoglycemic agents (e.g., glyburide, glimepiride); and diet and exercise therapy alone (as a control group).
  ○ Specification of metformin dosage and administration schedule.
  ○ Inclusion of studies investigating different metformin initiation timings (e.g., early vs. late in pregnancy).
- Outcomes:
  ○ Maternal Outcomes: rates of hypoglycemia, hyperglycemia, and glycemic control (e.g., HbA1c, fasting blood glucose, postprandial blood glucose), pregnancy-related complications (e.g., pre-eclampsia, gestational hypertension, preterm birth, cesarean section), maternal weight gain during pregnancy, and postpartum complications (e.g., postpartum hemorrhage, infection).
  ○ Neonatal Outcomes: birth weight (macrosomia, small for gestational age), neonatal hypoglycemia, respiratory distress syndrome, neonatal intensive care unit (NICU) admission and length of stay, and congenital anomalies.
- Publications: full-text articles published in peer-reviewed medical journals indexed in major databases (e.g., PubMed, Embase, Cochrane Library) and studies published in English.

Exclusion Criteria:

- Participants: women with pre-existing diabetes (type 1 or type 2), with contraindications to metformin use (e.g., hepatic or renal impairment, lactic acidosis, vitamin B12 deficiency), with significant medical comorbidities (e.g., severe cardiovascular disease, chronic kidney disease), with fetal abnormalities detected prior to study enrollment, and undergoing assisted reproductive technologies (ARTs).
- Study Design: case reports, case series, animal studies, and review articles, studies with significant methodological limitations (e.g., small sample size, lack of blinding, inadequate data collection), and studies with a high risk of bias.
- Outcomes: studies solely focusing on surrogate outcomes (e.g., insulin resistance markers) and studies with inadequate or incomplete reporting of outcomes.
- Publications: research published in case reports, conference abstracts, and/or guidelines.

### 2.4. Literature Screening and Data Extraction

The review followed the PRISMA requirements (Preferred Reporting Items for Systematic Reviews and Meta-Analyses) but was not registered [46]. The retrieved records were subjected to semi-automatic deduplication using Rayyan [47]. Following the first literature search, three authors evaluated the studies by examining the titles and abstracts. Any conflicts were handled by reaching a consensus or discussing with a fourth author. Excluded were publications that were not relevant, and the remaining complete copies of papers were evaluated for eligibility by two reviewers who were unaware of the details based on the PICOS criteria. Discrepancies were once again handled through consensus or with the assistance of a third reviewer. The references of the full-text publications were examined to mitigate the risk of overlooking relevant research not captured by the database search. All reviewer differences were resolved by engaging in discussions with a fourth reviewer, thus guaranteeing that no pertinent studies were omitted. The data extraction procedure collected essential details such as author biographies, publication years, intervention methodologies, outcome measurements, study designs, and classification approaches. The data items that were retrieved comprised the year of publication, study design, nation, center, study duration, number of participants, age, type of medicine for GDM, maternal outcomes, and neonatal outcomes including, among others, gestational weight gain (GWG), glycemic control (GC), pregnancy-induced hypertension (PIH), cesarian deliveries (CDs), gestational age at birth (GAB), gestational birth weight (GBW), neonatal intensive care unit (NICU) admission, large for gestational age (LGA), and small for gestational age (SGA).

### 2.5. Risk of Bias

The risk of bias was assessed using the Cochrane ‘risk of bias’ tool 2. We assessed the following items: random sequence generation, allocation concealment, blinding of participants, personnel and assessors, incomplete outcome data, and selective reporting. Studies were subsequently classified as being at an overall low risk of bias when all of these items were rated as low risk. Studies were classified as having some risk of bias when one item was rated as being of some concern. Studies were classified as being at a high risk of bias when they were judged to be at a high risk of bias in at least one domain or they were judged to have some concerns for multiple domains.

## 3. Results

**Figure 1:**
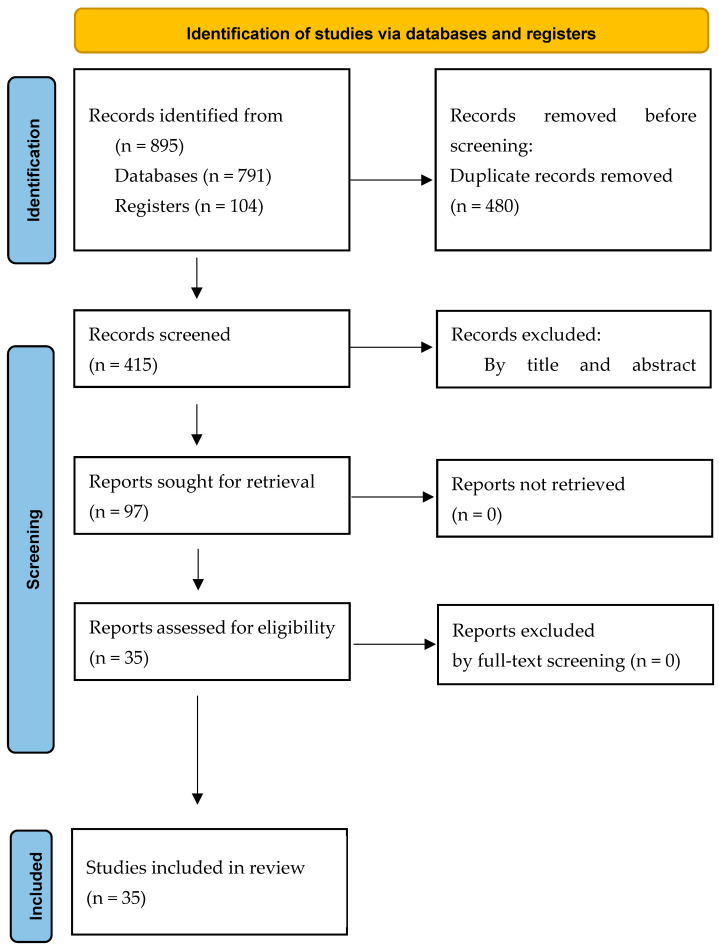
Flow diagram of the study design, literature search, study selection and data extraction process for this review.

The process of selecting the studies is outlined in Figure 1. At first, 895 papers were found, and 415 were considered suitable for further screening after deleting duplicates. Afterward, these 415 articles were subjected to a title–abstract examination, and out of those, 97 articles satisfied the inclusion requirements. After a comprehensive evaluation of the full text, 62 studies were deemed unsuitable and therefore excluded, whereas 35 papers met the criteria and were included in the current review. Of these, there were 19 randomized controlled trials (RCTs), while the remaining 16 were systematic reviews and meta-analyses (SRs/MAs). The results of the assessment of risk of bias for the RCTs are presented in Table 1.

In 2007, Moore et al. were the first to present preliminary data supporting the efficacy and safety of metformin as an insulin alternative for GDM [48]. They compared the glycemic concentrations in 63 women with GDM treated with metformin or insulin (n = 32 and n = 31, respectively). The two groups had no difference in their mean fasting and 2 h postprandial blood glucose concentrations. No patient discontinued metformin due to treatment failure and necessitated insulin therapy. The gestational age at admission and GAB were not different (*p* = 0.077), nor was the number of CD (*p* = 0.102) or the incidence of neonatal complications (*p* = 0.144–0.373) between the two groups.

In 2008, Rowan et al. [49] randomized 751 Australian women with GDM to receive either metformin (with supplementary insulin if needed) or insulin (n = 373 and n = 378, respectively). The study aimed to determine if there was a 33% increase in the composite outcome among newborns of mothers treated with metformin compared with those treated with insulin. Additional outcomes were neonatal physical assessments, maternal blood glucose regulation, maternal hypertension, postpartum glucose tolerance, and treatment satisfaction. In the metformin group, 92.6% took it until delivery, and 46.3% required additional insulin. This latter group was associated with a higher body mass index (BMI) (*p* = 0.01), higher initial fasting glucose concentrations (*p* < 0.001), higher hemoglobin A1c (HbA1c) concentrations (*p* < 0.001), higher incidence of miscarriages (*p* < 0.001), a lower frequency of nulliparous women (*p* = 0.003), and a higher percentage of Polynesian ethnicity (*p* < 0.001). Of the metformin-treated women, 76.6% would prefer their medication again in a future pregnancy, compared with only 27.2% of the insulin-treated ones (*p* < 0.001). There was no difference in the composite primary outcomes (*p* = 0.95). The metformin group was characterized by a lower incidence of severe neonatal hypoglycemia (*p* = 0.008) and preterm deliveries (*p* = 0.04). No differences existed in the frequencies of other secondary outcomes, and no negative effects were associated with metformin administration. In detail, the GAB, the mean maternal 2 h postprandial glucose concentrations, and the GWG were slightly lower in the metformin group than in the insulin group. In contrast, the relative weight loss between enrollment and the postpartum visit was higher (*p* = 0.006). The study was characterized by an extended enrolment time ranging from 20 to 33 gestational weeks, while the 2-year follow-up visit did not keep track of the potential lifestyle changes of the enrolled women. Despite these limitations, the study was the first to support the efficacy and safety of metformin for GDM pregnancies compared with the traditional insulin administration.

In 2011, Goh et al. compared the differences in maternal and neonatal parameters among metformin, insulin, and diet [50]. The authors prospectively analyzed the National Women’s Health Registry. Women with GDM (n = 1269) were included in three groups: diet changes (n = 371), insulin (n = 399) and metformin (n = 465) either alone or combined with insulin (n = 249 and n = 216, respectively). Women treated with medications had higher BMIs (*p* < 0.001) and higher fasting glucose upon diagnosis (*p* < 0.001) compared with women with a fixed diet plan. The insulin group had higher rates of CD (45.6%), preterm deliveries (19.2%), customized LGA infants (18.5%), NICU admissions (18.7%) and neonatal intravenous dextrose use (11.1%) compared with the corresponding rates of metformin and diet groups (37% and 34%, *p* = 0.02; 12.5% and 12.1%, *p* = 0.005; 12.5% and 12.4%, *p* = 0.02; 12.7% and 14%, *p* = 0.04; 5.1% and 7.4%, *p* = 0.004, respectively). Furthermore, women treated with a combination of metformin and insulin had increased BMI and fasting blood glucose concentrations during oral glucose tolerance tests (OGTTs) as well as a higher incidence of CD and preterm births compared with the insulin-only group (62.8% and 45.3%; 5.7 mmol/L and 5.4 mmol/L; 45.6% and 38%; 19.2% and 12.8%, respectively). The study concluded that fewer poor outcomes from the use of insulin accompanied the administration of metformin in GDM pregnancies. However, baseline variations between the different groups might have affected this result.

Another study was carried out in 2011 by Ijäs et al., evaluating whether metformin is as efficacious as insulin in preventing fetal macrosomia in GDM-affected pregnancies [51]. This Finnish open-label RCT enrolled 100 women with singleton pregnancies ranging from 12 to 34 gestational weeks with GDM who unsuccessfully tried to reach normal blood glucose concentrations via diet. Women were randomly assigned to insulin or metformin (50 in each group). The incidence of LGA newborns and neonatal morbidity were the main study outcomes, while neonatal complications, including NICU admission, neonatal traumas, or hypoglycemia were the secondary outcomes. The insulin and metformin groups had no difference in the incidence of LGA (8.5% and 10.0%, respectively, *p* = 0.97), mean GBW, mean arterial cord pH, or newborn morbidity. Some 15 out of the 47 women assigned to metformin required supplementary insulin; they had higher BMI (*p* = 0.002) and fasting blood glucose concentrations in OGTT (*p* = 0.001) and needed medical treatment for GDM earlier during their pregnancy (*p* = 0.002) compared with women treated with metformin only. Additionally, metformin was associated with a higher probability of CD than insulin [relative risk (RR) 1.9] and higher GBW at delivery (*p* = 0.022). The study demonstrated that metformin is effective in preventing fetal macrosomia, particularly in lean or moderately overweight women developing GDM in late gestation.

In 2012, Tertti et al. organized a single-site RCT to compare the efficacy of metformin and insulin in 217 patients with GDM using a non-inferiority design, with GBW as the main outcome [52]. The metformin and insulin groups showed no changes in mean GBW or other newborn and maternal data. Only 23 (20.9%) of 110 metformin-treated women required additional insulin. Compared with women on metformin alone, those requiring supplementary insulin were older (*p* = 0.04), had higher HbA1c (*p* = 0.01) and fructosamine (*p* < 0.001) at randomization, and had earlier OGTT examination (*p* = 0.01) and GDM therapy initiation (*p* = 0.004). Women with baseline blood fructosamine concentrations above the median were 4.6 times more likely to require extra insulin than those with concentrations below the median (*p* = 0.006). However, the relative risk for HbA1c between patients with HbA1c concentrations above and below the median value was not significant (*p* = 0.09), leading to the assumption that fructosamine may be a more useful predictor than HbA1c in determining the need for additional insulin.

Another study conducted in 2012 by Niromanesh et al. tried to assess the efficacy of metformin and insulin in women with GDM [53]. The study included 80 women who were treated with metformin and 80 women who were treated with insulin. All participants had singleton pregnancies between 20 and 34 gestational weeks. The women had similar maternal features and could not attain GC. The main outcomes were the mother’s ability to control her fasting blood glucose concentrations and the newborn’s GBW. Throughout GDM therapy, the two groups had identical mean fasting blood (*p* = 0.68) and postprandial (*p* = 0.87) glucose concentrations. The offspring from mothers in the metformin group had a lower incidence of >90 birth weight centile (*p* = 0.012) and decreased GBW (*p* < 0.001) compared with the insulin group. Eleven of the eighty metformin-treated women required additional insulin to achieve euglycemia. The study found no substantial risk of unfavorable maternal or neonatal outcomes with metformin compared with insulin.

In 2013, Spaulonci et al. carried out an RCT to compare metformin and insulin for GDM treatment for women who were unable to maintain glycemic control with diet and exercise and to discover characteristics that indicate the need for supplementary insulin in women who were initially treated only with metformin [54]. The sample was 94 women who were randomized between the two medications in a 1:1 ratio, with the enrollment criteria being singleton pregnancy, diet, exercise for at least one week without good glycemic management, lack of risk factors for lactic acidosis, absence of anatomic and/or chromosomal defects in the conceptus, and presence of prenatal follow-up. The mean pretreatment glucose concentrations comparison was not different between the two groups (*p* = 0.79). Following the drug’s administration, the metformin group had lower mean glucose concentrations (*p* = 0.02), mostly due to lower concentrations after supper (*p* = 0.042); gained less weight (*p* = 0.002); and had a decreased risk of neonatal hypoglycemia (*p* = 0.032). It is worth noting that 26% of the patients in the metformin group required additional insulin therapy. The study showed a correlation between the likelihood of not responding to metformin monotherapy and two factors: an earlier gestational age at diagnosis (*p* = 0.032) and a higher mean pretreatment glucose concentration (*p* = 0.046). Logistic regression analysis revealed that early gestational age at diagnosis and mean pretreatment glucose concentrations were predictors of the need for additional insulin therapy in women originally treated with metformin (*p* = 0.032 and 0.046, respectively).

The same year, Gui et al. performed a meta-analysis including five RCTs and 1270 pregnant women [67]. The metformin group experienced a reduced mean GWG (*p* = 0.003), a decreased mean GAB (*p* = 0.02), an increased incidence of preterm birth (*p* = 0.01) and a reduced incidence of PIH (*p* = 0.02) than the insulin group. Also, this group had lower OGTT fasting glucose concentrations (*p* = 0.0006) compared with the supplemental insulin group. This meta-analysis’ limitations were the inhomogeneity among the studies regarding the frequency of needing additional insulin, the metformin dosage, the criteria used to diagnose GDM, and the glycemic targets.

In 2014, Ruholamin et al. carried out an RCT including 109 women with GDM who did not achieve sufficient GC with dietary interventions [55]. The patients were administered either metformin or insulin. The results showed that the mean fasting blood sugar and postprandial readings were similar between the two groups. At the same time, there seemed to be no significant difference in pregnancy complications. When comparing the neonatal outcomes, there were no significant statistical differences between the groups in terms of incidence of hypoglycemia, hyperbilirubinemia, average GBW, fifth-minute Apgar score < 7 or umbilical artery pH < 7.05. The same year, Su and Wang designed a meta-analysis which included six RCTs with a total of 1420 participants [68]. Their findings indicated that the use of metformin in pregnant women with GDM did not have a substantial impact on negative outcomes for both the mother and the newborn. Additionally, it was associated with reduced GWG and neonatal hypoglycemia but a greater occurrence of premature birth.

In 2015, Ainuddin et al. designed an open-labeled RCT to compare the therapeutic indexes among metformin alone, insulin alone and their combined use (when metformin alone did not achieve glucose targets) in 150 women with GDM [56]. The metformin group had a lower GWG increase and mean birth weight compared with the two other groups (*p* < 0.001 and 0.01, respectively). Additionally, Zhao et al. published their meta-analysis of eight RCTs with 1592 total participants, which revealed that metformin exhibited statistically significant effects on PIH (RR 0.54) without having a significant impact, however, on respiratory distress syndrome, phototherapy, perinatal death, neonatal hypoglycemia and the incidence of LGA infants [69]. A similarly sized meta-analysis was carried out by Kitwitee et al., which comprised eight RCTs, including 1712 participants [70]. The combined estimates of the differences between metformin and insulin on fasting plasma glucose, postprandial plasma glucose, and HbA1c concentrations, assessed at 36–37 weeks of gestation, were minimal and statistically insignificant. Metformin administration was linked to a reduced occurrence of neonatal hypoglycemia (RR 0.74) and NICU admission (RR 0.76) compared with the insulin group. Bayesian analysis established that metformin consistently had greater effectiveness than insulin, with a probability exceeding 98%, in treating these two complications. There was no statistically significant difference in outcomes between the two treatment groups.

In the same year, Balsells et al. designed a meta-analysis of 15 studies and 2509 women with GDM to compare the efficacy of metformin, insulin, and glibenclamide [71]. The metformin group had lower maternal GWG and GAB and a lower incidence of preterm birth than the insulin group, with a trend for neonatal hypoglycemia. Compared to the glibenclamide group, the metformin group had lower GWG and GBW and a lower incidence of macrosomia. However, metformin was associated with greater treatment failure rates than glibenclamide. Furthermore, Singh et al. carried out a literature review including seven RCTs with a total sample size of 1514 women that concluded that most clinical trials reported no significant disparity in glycemic control between the metformin and insulin groups [72]. When comparing maternal outcomes, four trials found that those receiving metformin treatment had lower GWG. Multiple studies have documented a reduced incidence of newborn hypoglycemia, but one study has found an increased incidence of preterm birth in the metformin group.

In 2016, Zhu et al. performed a meta-analysis that included all available RCTs from 1946 to 2014, comparing the efficacy of metformin and insulin in GDM [73]. They synthesized data from 8 RCTs and 1712 women with GDM (853 treated with metformin and 859 with insulin). Metformin did not enhance the risk of preterm deliveries (*p* = 0.19). At the same time, it was associated with a reduction in total GWG, GWG after randomization, neonatal hypoglycemia episodes, and NICU admissions (*p* = 0.01, <0.00001, 0.0003 and 0.002, respectively). Finally, metformin was not associated with pre-eclampsia, CD, hyperbilirubinemia, and macrosomia incidences.

Similar findings were observed in the systematic review and meta-analysis conducted in 2017 by Butalia et al.; data from 16 studies comparing metformin vs. insulin as GDM therapies were included, forming a cohort of 2165 women [74]. The authors concluded that metformin administration against GDM was not linked to any short-term harm during pregnancy for the mother or the offspring.

Another meta-analysis by Feng and Yang [75] reported that maternal weight gain and HbA1c concentrations were considerably lower in the metformin group compared with the insulin group. Also, metformin minimized gestational hypertension problems in women with GDM, most likely by lowering endothelial activation and maternal inflammatory response to insulin resistance.

In 2018, Valdes et al. tried to clarify if metformin could be used as a GDM treatment in women with pregestational insulin resistance [57]. This double-blind, multicenter RCT involved 140 patients who were administered either 1700 mg of metformin per day (n = 68) or a placebo (n = 74). Patients were recruited between the 12th and 15th gestational weeks, with therapy continuing until the 36th gestational week. Metformin did not reduce the incidence of GDM compared with placebo (37.5% and 25.4%, respectively; *p* = 0.2). Furthermore, metformin was associated with an increase in medication intolerance as compared to placebo (14.3% and 1.8%, respectively; *p* = 0.02).

In the same year, a study by Eid et al. indicated that metformin considerably reduces overall weight increase and weight gain following randomization compared with insulin (*p* = 0.0001) [58]. Although the mean fasting and postprandial glucose concentrations during treatment and the HbA1c concentrations did not differ between the groups, the incidence of LGA and macrosomia in the insulin group was higher than in the metformin group.

In 2019, Guo et al. designed a meta-analysis to compare the efficacy and safety profile of the three main GDM therapies (insulin, metformin, and glyburide) [76]. The authors extracted data from 41 studies and 7703 women with GDM. Their findings revealed that the metformin-treated patients were associated with a lower GWG increase than glyburide (*p* < 0.05) and a lower GBW and GAB compared with insulin (*p* < 0.01). The meta-analysis concluded that metformin is a safe and effective therapy for GDM.

The same year, Gnomian et al. carried out their RCT, which consisted of 286 pregnant women with GDM. The participants were randomly allocated into two equal cohorts: insulin and metformin [59]. Serum fasting plasma glucose (FPG), 2 h plasma glucose (PG), and glycated hemoglobin (HbA1c) concentrations were measured bimonthly until childbirth. Additional variables captured in the study included birth delivery type, reason for cesarean section, gestational age at delivery, birth trauma, Apgar score, GBW, NICU admission, and newborn hypoglycemia. Following the completion of the treatment, there were no notable variations in FPG, PG, and HbA1c concentrations between the two groups, and no statistically significant differences were found between the two groups in terms of the birth delivery technique, CD, birth trauma, Apgar score, GBW, NICU admission, and neonatal hypoglycemia. Moreover, Landi et al.’s RCT from New Zealand studied 3818 pregnancies treated with metformin and 3450 pregnancies treated with insulin [71]. The two groups exhibited similar characteristics regarding age, BMI, and diagnosis/treatment initiation timing. Following correction, metformin showed a decreased absolute risk of planned CD [risk differences (RD) −2.3] and a significant risk for GAB (RD −3.7) and newborn hypoglycemia (RD −5.0) compared to hormone therapy. No clinically significant changes were observed in average GBW between pregnancies treated with metformin and insulin.

In 2020, 502 women were involved in an RCT in which 253 received metformin while the remaining 249 received a placebo [61]. Compared to the placebo group, women treated with metformin achieved better control of blood sugar levels and had less GWG (*p* < 0.0001) and fewer CDs (RR 0.85, *p* = 0.031). The study revealed no substantial disparity in PIH between the two groups. In the metformin group, compared to infants in the placebo group, infants exposed to metformin had a lower average GBW (*p* = 0.002), and fewer were above the 97th centile for birthweight (RR 0.58, *p* = 0.041) or were LGA (RR 0.65, *p* = 0.046).

In 2021, Bao et al. conducted a meta-analysis [77] including 24 studies, of which 17 were RCTs. Metformin reduced the risk of PID (*p* = 0.03), LGA babies (*p* = 0.04), macrosomia (*p* = 0.01), neonatal hypoglycemia (*p* = 0.001), and NICU admission (*p* = 0.01). Furthermore, metformin was not associated with an increase in the incidence of preterm deliveries (*p* = 0.11), pre-eclampsia (*p* = 0.45), CD (*p* = 0.20), and small-for-gestational-age (SGA) neonates (*p* = 0.95). The meta-analysis added value to the use of metformin in GDM pregnancies.

The same year, a more extended meta-analysis was conducted by Tarry-Adkins [78] to evaluate the effects of metformin on pregnancy. It synthesized 35 relative studies and 8033 women. Metformin-treated pregnancies were associated with reduced weight gain (*p* < 0.0001), decreased incidence of pre-eclampsia (*p* = 0.02), and increased incidence of gastrointestinal adverse effects (*p* = 0.0002). Another meta-analysis encompassing 32 studies and a collective sample size of 5964 patients concluded that compared to insulin, metformin demonstrated superior effectiveness in reducing the occurrence of macrosomia (RR 0.66, *p* = 0.005), lowering the rate of NICU admission (RR 0.78, *p* = 0.002), decreasing the incidence of neonatal hypoglycemia (RR 0.67, *p* < 0.0001), reducing GBW [standardized mean difference (SMD) −0.37, *p* = 0.004], lowering the occurrence of LGA (RR 0.76, *p* = 0.002), shortening GAB (mean difference (MD) −0.22, *p* = 0.0002), reducing GWG (MD −1.41, *p* = 0.001), decreasing the rate of CD (RR 0.86, *p* = 0.0004), and reducing the occurrence of PID (RR 0.47, *p* = 0.01) [79]. One smaller meta-analysis was conducted the same year by He et al. [80]. In the analysis of 21 studies and 4545 women with GDM, the metformin group was associated with reduced maternal weight gain, gestational age at birth, and birthweight (*p* < 0.00001, 0.02 and <0.0001, respectively) and lower incidence of gestational hypertension and hypoglycemia (*p* = 0.0006).

Another study was carried out in 2021 by Picón-César et al., aiming to verify whether metformin could achieve the same glycemic control as insulin, as well as equivalent obstetrical and perinatal outcomes, with a favorable safety profile, in women with GDM that could be optimized only by lifestyle changes [62]. This RCT was an open-label, parallel-arm study conducted at two hospitals in Málaga and enrolled 200 women aged 18 to 45 in the second or third trimester of singleton pregnancy between 2016 and 2019. The women were randomized between the two medications in a 1:1 ratio. Their gestational age was between 14 and 35 weeks, and they were diagnosed with GDM requiring pharmacologic treatment. The key outcome measures were glycemic management, encompassing mean blood glucose levels and incidence of hypoglycemia, as well as maternal and neonatal complications, including gestational hypertension, induced or spontaneous labor, preterm delivery, fetal growth abnormalities, NICU admission, respiratory distress syndrome, neonatal hypoglycemia, and the need for phototherapy for jaundice. The study validated the association between metformin and reduced maternal weight increase (*p* = 0.011). Additionally, no differences were observed in birth weight, SGA or LGA rates. The mean fasting and postprandial glycemia were similar across groups, although postprandial glycemia was lower after lunch or supper in the metformin-treated group. The insulin group had a higher incidence of hypoglycemic episodes (*p* < 0.001), labor inductions (*p* = 0.029), cesarean section (*p* = 0.001), and higher weight gain (*p* < 0.001) compared with the metformin group. The groups did not vary in terms of mean birthweight, macrosomia, or neonatal morbidity. Women treated with metformin had a reduced cesarean section rate, which was not connected with macrosomia, SGA, LGA, or any other pregnancy problems.

In 2022, the same Spanish team conducted an RCT to investigate the changes in gut microbiota composition and function among women with severe GDM treated with metformin or insulin [63]. This single-center study included 58 women, 30 of whom were treated with metformin and 28 with insulin, from the University Hospital of Málaga. The metformin group had reduced mean postprandial glycemia and a smaller increase in weight and BMI compared with the insulin group. The 16S rRNA analysis from stool samples reported a decrease in Firmicutes and Peptostreptococcaceae and an increase in Proteobacteria and Enterobacteriaceae in the metformin group. Negative associations were observed between changes in the abundance of Proteobacteria and average postprandial glycemia (*p* = 0.023). Similarly, inverse associations were detected between Enterobacteriaceae and increased BMI and weight gain (*p* = 0.031 and 0.036, respectively). The metformin group demonstrated an increased prevalence of metabolic pathways linked to propionate degradation and the production of ubiquinol within the gut microbiome’s profile. Metformin appears to exert its beneficial effects on glucose regulation by inducing alterations in the gut microbiome that may help to ameliorate the gut dysbiosis commonly observed in gestational diabetes mellitus, a condition characterized by inflammation, excessive adiposity, and impaired glucose metabolism [79,80,81]. Pregnancy-associated gut dysbiosis has been associated with several pathogenic bacteria belonging to the Firmicutes, Proteobacteria, Bacteroidetes, and Actinobacteria phyla, including Ruminococcaceae, Desulfovibrio, Enterobacteriaceae, P. distasonis, Prevotella, and Collinsella [81,82,83,84] Additionally, there is a reduction in the abundance of bacteria that produce butyrate, such as Faecalibacterium and Bifidobacterium [81,82,83,84]. Several studies have found that microbial dysbiosis in women with GDM resembles the gut microbiota patterns of women with T2DM [85,86,87]. The study from Molina-Vega et al. was pioneering since it triggered the search for other potential targets for metformin’s actions [88]. It could also explain why gastrointestinal adverse effects were higher in metformin-treated women than in different groups in the meta-analysis by Tarry-Adkins et al. [78].

In the same year, Li et al. collected data from 26 RCTs to conduct a meta-analysis comparing the efficacy and safety of metformin compared with insulin in 4921 GDM women [89]. Metformin was associated with lower overall risk estimates for pre-eclampsia, hypertension (*p* < 0.05), hypoglycemia (*p* < 0.05), and NICU admission (*p* < 0.05). However, the overall risk estimates for neonatal macrosomia and neonatal injury were higher (*p* < 0.05 and *p* < 0.01, respectively).

Another RCT was conducted at the University Hospital of Málaga in 2022 to assess the effect of metformin on HbA1c concentrations at 36 gestational weeks in women with GDM treated with fixed diet modification [64]. This RCT was a double-blind, placebo-controlled, single-center study in which 106 women with GDM participated at 16–30 gestational weeks and were assigned to either metformin or placebo until delivery. HbA1c concentrations were measured at the beginning of the study and again at 36 gestational weeks, with the main outcome being the alteration in HbA1c concentrations. During pregnancy, HbA1c concentrations rose considerably in both treatment groups (*p* < 0.001), with no difference between them (*p* = 0.310). The metformin group had a lower mean birth weight (*p* = 0.030) than the placebo group, while the rates of low birth weight were similar (*p* = 0.123 and *p* = 0.102, respectively) between the groups. The study concluded that metformin could not prevent the increase in HbA1c at 36 gestational weeks. The mean birth weight was lower, which was alarming.

In 2023, Dunne et al. carried out an RCT in Ireland to determine whether the early initiation of metformin at gestation weeks 32 or 38 results in a reduction in insulin initiation or an improvement in baseline hyperglycemia [66]. The study included 510 individuals (535 pregnancies) who were diagnosed with GDM and were randomized 1:1 to either placebo or metformin (maximum dose, 2500 mg) in addition to standard care. The primary outcome was a composite of a fasting glucose level of 5.1 mmol/L or higher at gestation weeks 32 or 38 or the initiation of insulin which was not significantly different between the two groups. It occurred in 150 pregnancies (56.8%) in the metformin group and 167 pregnancies (63.7%) in the placebo group. Time to insulin initiation, self-reported capillary GC, and GWG were three of the six prespecified secondary maternal outcomes that favored the metformin group. There were differences in secondary neonatal outcomes between the two groups. The metformin group had smaller neonates (lower MBW, a lower proportion weighing >4 kg, a lower proportion in the >90% percentile, and a smaller crown-heel length). However, there were no differences in NICU admissions, respiratory distress requiring respiratory support, jaundice requiring phototherapy, major congenital anomalies, neonatal hypoglycemia, or the proportion with 5-min Apgar scores < 7.

In 2024, Wu et al. performed a meta-analysis including 24 RCTs and a total sample of 4934 GDM participants [65]. Compared with insulin, metformin demonstrated a significant reduction in the risks of pre-eclampsia (RR 0.61), induction of labor (RR 0.90), CD (RR 0.91), macrosomia (RR 0.67), NICU admission (RR 0.75), neonatal hypoglycemia (RR 0.55), and LGA infants (RR 0.80, 95% CI 0.68 to 0.94, *p*  =  0.007). Conversely, metformin showed no significant impact on gestational PIH, spontaneous vaginal delivery, emergency cesarean section, shoulder dystocia, premature birth, polyhydramnios, birth trauma, 5-min Apgar score < 7, being small for gestational age (SGA), respiratory distress syndrome (RDS), jaundice, or congenital defects. The same year, Paschou et al. conducted an SR in which metformin’s efficacy in managing GDM was well established, as it effectively controlled blood sugar levels and reduced the risk of complications. This work delved into metformin’s pharmacokinetics during pregnancy, highlighting its transplacental transmission and potential impact on fetal growth and metabolism. The need for additional insulin alongside metformin was also discussed, with factors like patient BMI and baseline glycemic control influencing this requirement [90].

Metformin’s safety during pregnancy, especially concerning fetal development, is a key focus of ongoing research. While studies show its safety in early gestation, the long-term effects are still being investigated. Table 2 summarizes the short-term outcomes (maternal and neonatal) of metformin use during pregnancy in all the studies presented in this section.

## 4. Discussion

In the last two decades, the literature has provided evidence that metformin can effectively treat GDM in women who fail to achieve optimal glucose control through diet and exercise. It is worth noting that in several studies, approximately 50% of women treated with metformin required insulin therapy to achieve glycemic targets. This study’s principal objective was to present all the available research that assesses the effectiveness and safety of metformin against insulin and other drugs in treating GDM pregnancies. A comprehensive literature review was conducted, followed by a rigorous selection procedure using predetermined inclusion and exclusion criteria, resulting in 35 studies. An array of neonatal and maternal short-term outcomes were evaluated. All included studies are of relatively high quality, with no substantial variation in study outcomes and no overt publication bias detected.

In many studies, either RCTs or SRs/MAs, metformin was demonstrated to be an effective agent for obtaining glycemic control in women with GDM [52,54,63,73,75,90]. However, multiple studies found no significant difference in fasting and postprandial glucose levels between metformin and insulin groups [48,53,55,59,62]. Additionally, gestational weight gain was shown to be substantially reduced in the metformin group between the time of diagnosis of GDM and delivery, as well as between the initiation of drug treatment and delivery [23,50,53,54,56,57,63,64,72,73,74,75,77,78]. Concerning other maternal outcomes, such as pregnancy-induced hypertension and cesarian deliveries, the literature was inconsistent with other studies showing an association between metformin administration and lower incidence of both conditions [23,53,62,74,75,77,80,90]. One study showed lower risk estimates for hypertension and pre-eclampsia in the metformin group [89] while in another study, increased gastrointestinal adverse effects were observed in the metformin group [78]. Some studies reported a higher incidence of cesarean deliveries in the metformin group [50,51], while others found a lower incidence or no significant difference at all [60,61,62,89]. In contrast, other studies failed to reveal a significant association between metformin and other drugs [48,49,52,54,56].

Regarding short-term neonatal outcomes, most studies concluded that gestational age at birth does not seem to be affected differently in the metformin group [48,51,52,53,54,57,73,74,75]. In fact, some studies reported lower birth weight or a decreased incidence of LGA in the metformin group [53,56,61,71,89], but on the other hand, similar studies found no significant difference [51,52,59,60,62,66]. Many studies reported a lower incidence of neonatal hypoglycemia [49,54,62,68,70,72,73,89] and preterm deliveries [49,50] in the metformin group. Compared with other medications, the risks of increased gestational birth weight and NICU admission were statistically significantly lower in the metformin group [50,56,73,74,76,77,80,89].

Nevertheless, these findings need substantiation from more RCTs, most of which have a small sample size. Furthermore, there is a need for clinical trials specially tailored to assess the efficacy and safety of the combined administration of metformin and insulin. The data about their concurrent administration were derived from studies that compared metformin to insulin or from retrospective data collected from non-RCTs. The EMERGE trial is a phase III placebo-controlled RCT that was finalized at the end of 2023, aiming to investigate early metformin administration’s efficacy and safety profile as a GDM treatment [66,91]. In the same direction, the Metabolic Analysis for Treatment Choice in Gestational Diabetes Mellitus (MATCh-GDM) study aims to examine a personalized therapy strategy for GDM [92].

Nevertheless, as shown in the study by Yu et al., which analyzed data from 1998 to 2017 from the UK National Registry, metformin has been administrated for GDM treatment [73] over the last two decades. Metformin prescriptions climbed from <5% of GDM pregnancies before 2007 to 42.5% in 2008 and doubled in 2015, reaching an astonishing 85%.

Metformin achieves its therapeutic action by increasing glucose uptake in the liver and peripheral tissues and insulin sensitivity while reducing the need for glucose synthesis and altering the gut microbiota patterns. It is a cost-efficient substance with a low likelihood of hypoglycemia, and it does not require educational initiatives or stringent glycemic management, making it the preferable option, especially in rural and low-resource environments [45,77]. The potential risks associated with excessive weight during pregnancy, such as complications such as pre-eclampsia and high rates of cesarean delivery, may render the reduction in weight gain particularly advantageous. Additionally, metformin’s promising safety profile extends to the progeny; it does not appear to present substantial risks of adverse outcomes, including neonatal hypoglycemia or birth defects, when administered during pregnancy [49,54,68,70,72,73,80,89]. Metformin is a compelling consideration in the management of GDM due to its potential to enhance both maternal and neonatal health outcomes [90].

The main concern about using metformin as GDM treatment is its ability to cross the placenta, reach the fetal circulation, and affect neonatal development in later life [30,76,78]. A few hours after the mother administers metformin to the neonate, it is exposed to elevated levels [93,94,95,96]. This elevated exposure to metformin may be attributed to the fact that it is predominantly excreted through the renal route in adults. However, it is excreted into the amniotic fluid in the fetus, where it may be reabsorbed by the fetus and reintroduced into their circulation [7]. Consequently, it presents a potential issue due to the scarcity of knowledge on the subject, as there has been insufficient research focused on the metabolism of metformin in the neonate. Another area of concern is the correlation between metformin and an elevated risk of preterm births and reduced birth weights, although the severity of this effect may differ depending on the rationale behind its administration [49,50]. Additionally, research indicates that metformin may modify fetal programming, which could result in later-life predispositions to adolescent obesity and metabolic syndrome [90]. Therefore, in cases of metformin administration, clinicians should provide proper guidance to women, explaining all the risk-related information to them and their neonates.

An important aspect of this research was its thorough examination of the literature. This was accomplished by incorporating the most recent review on the subject, 35 studies from the international literature, and a comparison of three drugs (metformin, glyburide, and insulin) used to treat patients with GDM. There are certain limitations of this systematic review that merit careful consideration. Initial consideration should be given to the potential influence of the relatively small sample sizes in the included studies on the generalizability of the results. Additionally, the restriction to English-language studies may result in language bias, which could lead to the exclusion of pertinent research published in languages other than English. In addition, this review focused on the short-term results of patients and not the long-term results (through neonatal follow-up).

It is important to acknowledge that the findings of this review may not fully capture the complete picture of managing GDM. Future research should aim to address these limitations by considering studies published in languages other than English. Long-term follow-up studies are also needed to provide a more comprehensive understanding of the impact of treatment on patients with GDM. Additionally, future research should consider including diverse populations to ensure that findings are applicable across different demographics. Long-term follow-up studies could also help to determine the effectiveness of treatment strategies over an extended period of time.

Incorporating qualitative research methods could also provide valuable insights into the experiences and perspectives of patients with GDM, enhancing the overall understanding of the condition and its management. Furthermore, exploring the potential role of technology and telemedicine in improving access to care and monitoring outcomes for patients with GDM could be a promising avenue for future research. Moreover, collaboration between healthcare professionals from different specialties, such as obstetrics, endocrinology, and nutrition, could lead to more comprehensive and personalized approaches to managing GDM. While technology and telemedicine may improve access to care for some patients with GDM, it may not be accessible or suitable for all individuals, potentially exacerbating health disparities. Additionally, lifestyle interventions may not always be effective in managing GDM, as genetic and other factors can play a significant role in the development and progression of the condition. Therefore, a holistic approach that considers both medical and lifestyle factors is essential in effectively managing GDM. By addressing the complex interplay of genetic, environmental, and lifestyle factors, healthcare professionals can provide more tailored and effective treatment plans for individuals with GDM.

## 5. Conclusions

Our review emphasized metformin’s exceptional effectiveness and safety characteristics against GDM. Metformin presents itself as a potentially effective oral substitute to insulin in the treatment of GDM, demonstrating its capacity to regulate blood sugar levels, prevent weight gain during pregnancy, and decrease the occurrence of neonatal hypoglycemia. In light of these results, metformin is a potentially effective method for enhancing the safety and general health of pregnant women with GDM, thereby presenting a useful avenue for improving neonatal and maternal outcomes.

## Figures and Tables

**Table 1:** Risk of bias assessment using risk of bias tool 2 (ROB 2).

	1	2	3	4	5	Overall
Moore 2007 [48]	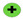	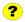	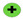	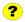	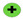	High
Rowan 2008 [49]	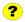	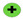	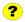	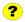	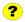	High
Goh 2011 [50]	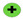	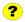	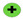	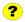	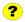	High
Ijäs 2011 [51]	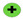	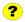	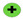	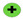	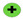	Some Concerns
Tertti 2012 [52]	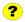	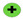	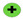	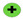	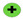	Some Concerns
Niromanesh 2012 [53]	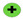	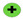	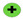	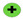	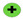	Low
Spaulonci 2013 [54]	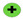	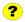	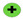	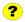	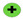	High
Ruholamin 2014 [55]	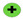	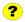	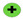	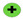	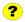	High
Ainuddin 2015 [56]	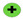	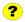	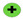	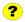	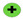	High
Valdes 2018 [57]	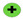	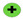	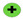	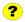	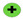	Some Concerns
Eid 2018 [58]	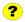	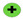	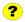	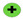	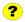	High
Ghomian 2019 [59]	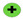	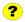	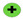	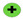	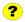	High
Landi 2019 [60]	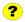	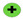	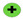	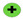	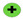	Some Concerns
Feig 2020 [61]	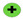	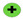	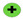	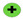	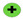	Low
Picón-César 2021 [62]	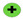	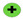	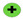	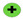	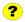	Some Concerns
Molina-Vega 2022 [63]	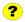	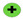	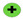	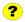	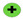	High
Tew 2022 [64]	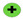	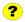	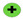	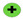	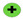	Some Concerns
Wu 2023 [65]	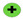	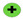	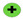	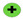	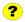	Some Concerns
Dunne 2023 [66]	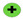	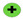	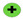	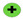	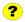	Some Concerns

1: Bias arising from the randomization process. 2: Bias due to deviations from intended interventions (effect of assignment to intervention). 3: Bias due to missing outcome data. 4: Bias in measurement of the outcome. 5: Bias in selection of the reported result.

**Table 2:** Summary of short-term outcomes (maternal and neonatal) of metformin use during pregnancy in all the presented studies compared to other medications.

Study	Year	Type	Intervention	Control	Key Outcomes
Moore et al. [48]	2007	RCT	MET	INS	Fasting and postprandial blood glucose levels were similar between the two treatment groups. No patient required INS after starting MET. There were no significant differences in gestational age at enrollment, CD, or neonatal outcomes such as GBW, Apgar scores, RDS, hyperbilirubinemia, GC, and NICU admission between the MET and INS groups.
Rowan et al. [49]	2008	RCT	MET (±INS)	INS	Of the 363 women prescribed MET, 92.6% continued taking it until delivery, and 46.3% also required INS. The primary composite outcome rate was 32.0% in the MET group and 32.2% in the INS group. There were no significant differences in secondary outcome rates between the groups. No serious adverse events were associated with MET use.
Goh et al. [50]	2011	RCT	MET (±INS)	INS/D	Women treated with MET and/or INS had significantly higher GWG and higher fasting glucose levels at diagnosis compared to those in the D group. Women treated with INS had higher rates of CD, preterm births, LGA infants, NICU admissions, and neonatal intravenous dextrose use compared to women receiving MET or D. Neonatal outcomes were similar between the D and MET treatment groups.
Ijäs et al. [51]	2011	RCT	MET (±INS)	INS	Neonatal outcomes were similar between the INS and MET groups. Some 15 of the 47 women randomized to MET required supplemental INS. These women were more obese, had higher fasting blood glucose levels, and required earlier GDM treatment than women with MET alone. There was a trend towards higher rates of CD in the MET group compared with the INS group.
Tertti et al. [52]	2012	RCT	MET (±INS)	INS	No significant differences were found in GBW between the MET and INS groups. There were no significant differences in neonatal or maternal outcomes between the groups. Only 23 of the 110 patients in the MET group required additional INS.
Niromanesh et al. [53]	2012	RCT	MET (±INS)	INS	The maternal characteristics were comparable between the two groups. Mean fasting and postprandial GC were similar throughout the GDM treatment. The MET group had a lower rate of infants with GBW above the 90th percentile compared to the INS group. GWG was reduced in the MET group. The two groups had similar rates of neonatal and obstetric complications. In the MET group, 14% of women required supplemental INS to achieve euglycemia.
Spaulonci et al. [54]	2013	RCT	MET (±INS)	INS	Results showed no significant difference in mean pretreatment GC between groups. However, after starting the medication, the MET group had lower GC, particularly after dinner. Women treated with MET had less GWG. Twelve women in the MET group required additional INS. Predictors of extra INS were earlier gestational age at diagnosis and higher mean pretreatment glucose levels.
Gui et al. [67]	2013	SR/MA	MET (±INS)	INS	The MET group had much lower GWG, significantly lower average GAB, higher incidence of preterm birth, and significantly lower incidence of PIH, compared to the supplemental INS group. The fasting blood sugar levels from the OGTT were significantly lower in the MET-only group than in the supplemental INS group.
Ruholamin et al. [55]	2014	RCT	MET	INS	The results showed that the mean fasting blood sugar and postprandial readings were similar between the two groups, and there were no significant differences in pregnancy complications. Comparing the neonatal outcomes, there were no statistically significant differences between the groups in the incidence of hypoglycemia, hyperbilirubinemia, average GBW, fifth-minute Apgar score < 7.00, or umbilical artery pH < 7.05.
Su & Wang [68]	2014	SR/MA	MET	INS	The use of MET did not substantially increase adverse maternal and neonatal outcomes. Additionally, there was a lower incidence of GWG and neonatal hypoglycemia, but there was a higher incidence of premature birth.
Ainuddin et al. [56]	2015	RCT	MET (±INS)	INS	The MET-treated groups had lower GWG and lower incidence of pre-eclampsia. Mean GBW was significantly lower in the MET-treated groups. Less neonatal morbidity was observed in the MET groups. Some 42.7% of patients in the MET group required supplemental INS, which was added at a mean age of 31.8 ± 5.9 gestational weeks.
Zhao et al. [69]	2015	SR/MA	MET	INS	MET had statistically significant effects in reducing the incidence of PIH. However, its effects on neonatal hypoglycemia, LGA infants, RDS, phototherapy, and perinatal death were not significant.
Kitwitee et al. [70]	2015	SR/MA	MET (±INS)	INS	The aggregated estimates of MET and INS differences were statistically non-significant and very small in fasting plasma glucose, postprandial plasma glucose, and HbA1c at 36–37 gestational weeks. MET treatment was associated with a lower incidence of neonatal hypoglycemia (RR 0.74) and NICU admission (RR 0.76) compared to the INS group.
Balsells et al. [71]	2015	SR/MA	MET (±INS)	INS/GL	Compared to INS, GL was associated with lower GBW, lower rates of LGA, and reduced neonatal hypoglycemia. MET compared to INS resulted in lower GBW, earlier GAB, and lower preterm birth rates, with a trend towards reduced neonatal hypoglycemia. When comparing MET to GL, MET was associated with lower GWG, lower GBW and fewer LGA infants. Four secondary outcomes were more favorable with MET compared to INS, while one outcome was less favorable with MET compared to GL. Notably, treatment failure was more common with MET than with GL.
Singh et al. [72]	2015	SR/MA	MET	INS	Most studies found no difference in GC between the MET and INS groups. When comparing maternal outcomes, women receiving MET had less GWG in four studies. Several studies reported lower rates of neonatal hypoglycemia with MET, while one study found higher preterm birth rates.
Zhu et al. [73]	2016	SR/MA	MET (±INS)	INS	MET did not increase the risk of preterm birth and was associated with reduced GWG. There were no significant differences in the rates of pre-eclampsia or CD between the groups. Importantly, MET significantly decreased the risk of neonatal hypoglycemia and NICU admission.
Butalia et al. [74]	2017	SR/MA	MET (±INS)	INS	The evaluation of sixteen studies revealed that MET reduced the risk of neonatal hypoglycemia, LGA babies, PIH, and total GWG. MET did not increase preterm delivery or CD.
Feng & Yang [75]	2017	SR/MA	MET (±INS)	INS	The rates of LGA infants, CD, neonatal RDS, and preterm births were similar between the MET and INSE groups. MET-group had better GC, while GWG and the incidence of PIH were lower.
Valdes et al. [57]	2018	RCT	MET	PL	MET administration did not reduce the incidence of GDM compared to PL. Additionally, MET was associated with significantly more drug intolerance than PL.
Eid et al. [58]	2018	RCT	MET	INS	MET significantly reduced GWG compared to INS. Although GC and HbA1c levels were similar between the groups, the INS group had a higher rate of LGA infants and macrosomia.
Guo et al. [76]	2019	SR/MA	MET (±INS)	INS/GL	Compared to MET, INS had a significantly higher risk of pre-eclampsia, NICU admission, neonatal hypoglycemia, and macrosomia and higher GBW and GAB compared to MET. MET was associated with lower GWG compared to GL.
Ghomian et al. [59]	2019	RCT	MET	INS	The results showed no significant differences between the MET and INS groups in maternal age, BMI, family history of diabetes, prior GDM, parity, fasting plasma glucose, 1 h and 2 h postprandial glucose, or 75 g OGTT before treatment. Similarly, there were no significant differences in fasting plasma glucose, plasma glucose, or HbA1c after treatment completion. The two groups also did not differ significantly in terms of delivery method, CD, birth trauma, Apgar scores, GBW, NICU admission, or neonatal hypoglycemia.
Landi et al. [60]	2019	RCT	MET	INS	The MET and INS groups were similar in age, BMI, and timing of diagnosis and treatment. After adjusting, MET was linked to lower risks of planned CD, LGA infants, and neonatal hypoglycemia compared to INS. There were no significant differences in average GBW between the two groups.
Feig et al. [61]	2020	RCT	MET	PL	There was no significant difference in the primary composite neonatal outcome between the two groups. Compared to the PL group, women treated with MET achieved better GC, required less insulin, gained GWG, and had fewer CD. There was no significant difference in PIH between the groups. Compared to the PL group, MET-exposed infants had lower GBW, fewer were above the 97th percentile for GBW, and fewer were LGA. These infants also had reduced adiposity measures. More infants in the MET group were SGA compared to the PL group, but there was no significant difference in cord C-peptide.
Bao et al. [77]	2021	SR/MA	MET (±INS)	INS	Compared to other treatments, MET was associated with reduced risk of PIH, LGA infants, macrosomia, neonatal hypoglycemia, and NICU admission, while it did not increase the risk of preterm birth, pre-eclampsia or CD.
Tarry-Adkins et al. [78]	2021	SR/MA	MET (±INS)	INS	GWG was lower in MET group compared to other treatments. MET was also associated with a reduced risk of pre-eclampsia. For other maternal outcomes assessed, the risk did not differ significantly between the groups.
Wang et al. [79]	2021	SR/MA	MET (±INS)	I/G	Compared to INS, MET was associated with lower rates of macrosomia, NICU admissions, neonatal hypoglycemia, higher GBW, SGA babies, earlier GAB, lower GWG, fewer CD, lower maternal postprandial blood glucose, and lower rates of PIH. However, GL, compared to INS, was associated with higher GBW and increased neonatal hypoglycemia. Meanwhile, MET, compared to GL, was associated with higher maternal fasting blood glucose but lower rates of labor induction.
He et al. [80]	2021	SR/MA	MET (±INS)	INS	Compared to INS, MET was associated with significantly lower risks of GWG, longer GAB, PIH, maternal hypoglycemia, higher GBW, neonatal hypoglycemia, NICU admission, macrosomia and LGA infants.
Picón-César et al. [62]	2021	RCT	MET (±INS)	INS	Women treated with MET had similar mean fasting and postprandial GC compared to the INS-treated group but had significantly better postprandial glycemia after lunch and dinner. The MET group also experienced fewer hypoglycemic episodes and lower GWG, and lower rates of labor induction and CD, without differences in mean GBW or other infant complications.
Molina-Vega et al. [63]	2022	RCT	MET (±INS)	INS	Compared to INS, women taking MET had lower mean postprandial GC and lower GWG.
Li et al. [89]	2022	SR/MA	MET (±INS)	INS	Compared to MET, INS was associated with a significantly higher risk of pre-eclampsia, PID, maternal and neonatal hypoglycemia, and NICU admission. However, the risk of neonatal macrosomia was lower with INS compared to MET.
Tew et al. [64]	2022	RCT	MET (±INS)	PL	HbA1c levels increased during pregnancy, with a mean rise of 0.20% ± 0.31% in the MET group compared to 0.27% ± 0.31% in the other group. Mean BWG was significantly lower in the MET group. However, the rates of macrosomia and low GBW were not significantly different between groups.
Wu et al. [65]	2023	RCT	MET (±INS)	INS	Compared with INS, MET demonstrated a significant reduction in the risks of pre-eclampsia (RR 0.61), induction of labor (RR 0.90), CD (RR 0.91), macrosomia (RR 0.67), NICU admission (RR 0.75), neonatal hypoglycemia (RR 0.55), and LGA infants (RR 0.80). Conversely, MET showed no significant impact on gestational PIH, spontaneous vaginal delivery, premature birth, 5-min Apgar score < 7, SGA infants, and RDS.
Dunne et al. [66]	2023	RCT	MET (±INS)	PL	The primary outcome was a composite of the initiation of insulin or a fasting glucose level of 5.1 mmol/L or higher at gestation weeks 32 or 38, which did not differ significantly between the two groups. It transpired in 150 pregnancies (56.2%) in the MET group and 167 pregnancies (63.7%) in the PL group. Three of the six prespecified secondary maternal outcomes that favored the MET group were time to insulin initiation, self-reported capillary GC, and GWG. The two groups exhibited disparities in secondary neonatal outcomes. The neonates in the MET group were smaller (with a lower MBW, a lower proportion of neonates weighing > 4 kg, a lower proportion of neonates in the >90% percentile, and a shorter crown-heel length). Nevertheless, there were no disparities in the proportion of neonates with 5-min Apgar scores < 7, with RDS, with need for phototherapy for jaundice or with neonatal hypoglycemia.
Paschou et al. [90]	2024	SR/MA	MET (±INS)	INS/GL/PL	MET was shown to be effective and safe, providing GC comparable to INS while reducing maternal GWG and risk of PIH. MET did not increase the risk of congenital abnormalities or other major adverse effects, such as low Apgar scores, NICU admissions, or RDS. MET was associated with increased preterm births and lower GBW.

CDs: cesarian deliveries, D: diet, GAB: gestational age at birth, GBW: gestational birth weight, GC: glycemic control, GL: glibenclamide, GWG: gestational weight gain, INS: insulin, LGA: large for gestational age, MET: metformin, NICU: neonatal intensive care unit, OGTT: oral glucose tolerance test, PIH: pregnancy-induced hypertension, PL: placebo, RCT: randomized control trial, RDS: respiratory distress syndrome; RR: relative risk, SGA: small for gestational age, SR/MA: systematic review/meta-analysis.

## Data Availability

This study did not create or analyze new data, and data sharing does not apply to this article.
